# Efficacious and Safe Tissue-Selective Controlled Gene Therapy Approaches for the Cornea

**DOI:** 10.1371/journal.pone.0018771

**Published:** 2011-04-12

**Authors:** Rajiv R. Mohan, Sunilima Sinha, Ashish Tandon, Rangan Gupta, Jonathan C. K. Tovey, Ajay Sharma

**Affiliations:** 1 Harry S. Truman Veterans Memorial Hospital, Columbia, Missouri, United States of America; 2 Mason Eye Institute, School of Medicine, University of Missouri, Columbia, Missouri, United States of America; 3 College of Veterinary Medicine, University of Missouri, Columbia, Missouri, United States of America; Johns Hopkins School of Medicine, United States of America

## Abstract

Untargeted and uncontrolled gene delivery is a major cause of gene therapy failure. This study aimed to define efficient and safe tissue-selective targeted gene therapy approaches for delivering genes into keratocytes of the cornea *in vivo* using a normal or diseased rabbit model. New Zealand White rabbits, adeno-associated virus serotype 5 (AAV5), and a minimally invasive hair-dryer based vector-delivery technique were used. Fifty microliters of AAV5 titer (6.5×10^12^ vg/ml) expressing green fluorescent protein gene (GFP) was topically applied onto normal or diseased (fibrotic or neovascularized) rabbit corneas for 2-minutes with a custom vector-delivery technique. Corneal fibrosis and neovascularization in rabbit eyes were induced with photorefractive keratectomy using excimer laser and VEGF (630 ng) using micropocket assay, respectively. Slit-lamp biomicroscopy and immunocytochemistry were used to confirm fibrosis and neovascularization in rabbit corneas. The levels, location and duration of delivered-GFP gene expression in the rabbit stroma were measured with immunocytochemistry and/or western blotting. Slot-blot measured delivered-GFP gene copy number. Confocal microscopy performed in whole-mounts of cornea and thick corneal sections determined geometric and spatial localization of delivered-GFP in three-dimensional arrangement. AAV5 toxicity and safety were evaluated with clinical eye exam, stereomicroscopy, slit-lamp biomicroscopy, and H&E staining. A single 2-minute AAV5 topical application via custom delivery-technique efficiently and selectively transduced keratocytes in the anterior stroma of normal and diseased rabbit corneas as evident from immunocytochemistry and confocal microscopy. Transgene expression was first detected at day 3, peaked at day 7, and was maintained up to 16 weeks (longest tested time point). Clinical and slit-lamp eye examination in live rabbits and H&E staining did not reveal any significant changes between AAV5-treated and untreated control corneas. These findings suggest that defined gene therapy approaches are safe for delivering genes into keratocytes *in vivo* and has potential for treating corneal disorders in human patients.

## Introduction

The success of gene therapy to treat diseases in human patients was first demonstrated over a decade ago [1]. Recent studies reporting significant improvement in vision with gene therapy in adult patients with Leber's congenital amaurosis affirmed the promise of gene therapy to treat eye diseases and prevent blindness in humans [2], [3]. In spite of the progress in gene therapy research, many challenges including the severe side effects caused by the vector and untargeted gene transfer remain to be resolved [4]–[6]. The success in the restoration of vision with gene therapy by curing retinal disorders has encouraged more research for defining gene therapy modalities for other ocular tissues. The potential of gene therapy to treat corneal disease has been investigated using various animal and *in vitro* models [7]–[13]. The cornea is an attractive organ for gene therapy because of its accessibility, immune-privileged status and ability to be monitored visually. The three major cellular layers of the cornea are: epithelium, stroma and endothelium. Gene therapy reagents can be administered into epithelium and stroma topically, as well as into stroma and endothelium with simple surgical procedures such as microinjection [13].

Major benefits of gene therapy are that it repairs the cause of the problem and not merely suppress symptoms, provides long-term cure, does not require repeated application or clinic visits. Various viral and non-viral vectors have been tested to deliver genes in the cornea [11]–[13]. Among viral vectors, adenoviruses and retroviruses have been shown to deliver genes into the cornea for short periods of time with moderate-to-severe inflammatory responses [14]–[20]. However, both of these vectors are of limited use for corneal gene therapy because of their inability to transduce non-dividing cells, low transduction efficiency for corneal cells and induction of immune reactions [12], [13]. Adeno-associated virus (AAV) and disabled lentivirus vectors offer better alternatives for delivering genes into the corneal stroma and endothelium because of their ability to transduce non-dividing cells [12], [13]. Additionally, these vectors are non-pathogenic and typically drive long-term transgene expression. AAV vectors are preferred over lentivirus because of their superior safety profile and non-pathogenicity to humans. More than 100 serotypes of AAV are known but serotypes 1-9 have been extensively tested for gene therapy [4], [11]–[13], [21]. AAV serotypes have shown a varied degree of tissue selective tropism [21]–[27]. These reports led us to the hypothesis that vector regulates amount of gene delivery in the cornea. Indeed our recent studies supported our hypothesis as AAV serotypes 2, 5, 6, 8, and 9 showed significantly different transduction in the rodent and rabbit cornea *in vitro* and *in vivo* [11], [21], [27], [28]. Our studies also suggested that AAV serotypes 5, 8 and 9 are most efficient for transporting genes in the rodent and rabbit stroma *in vivo* among various tested AAV serotypes [11], [21], [27]. AAV5-treated rodent corneas continued to express delivered transgene up to 1 year *in vivo* without any apparent side effects (Mohan et al Unpublished data), and thus was selected for this study.

The poor targeted delivery of therapeutic genes into corneal cells *in vivo* is another major challenge that sharply limits clinical application of gene therapy to treat corneal disorders and diseases. Previously, we demonstrated that AAV and plasmid vectors could deliver significant amount of genes into keratocytes of the rabbit stroma *in vivo* if applied on bare stroma employing a lamellar flap technique [28]. This led us to hypothesize that administration of an efficient vector via a custom vector-delivery technique would provide tissue-selective targeted transgene delivery in the cornea with no major side effects. Thus, multiple minimally invasive vector-delivery techniques to administer vector into keratocytes, stroma or endothelium of the rabbit and rodent cornea *in vivo* were optimized. Among many defined vector-delivery techniques, the hair-dryer based technique manipulating corneal hydration, the microinjection techniques using glass needle and Hamilton microsyringes, the topical cloning cylinder based technique employing 20% alcohol and the epithelial scrape technique using #64 surgical blade have provided the most targeted gene delivery into the targeted cells of the cornea *in vivo* [29]. The aim of this study was to define site-selective tissue-targeted gene therapy approaches using a suitable combination of efficacious AAV5 vector and newly-defined vector-delivery techniques to express therapeutic genes selectively in keratocytes or the stroma of the normal and damaged (fibrotic or neovascularized) rabbit cornea *in vivo.*

## Results

### Characterization of AAV5-mediated gene transfer in rabbit cornea

**Figure 1:**
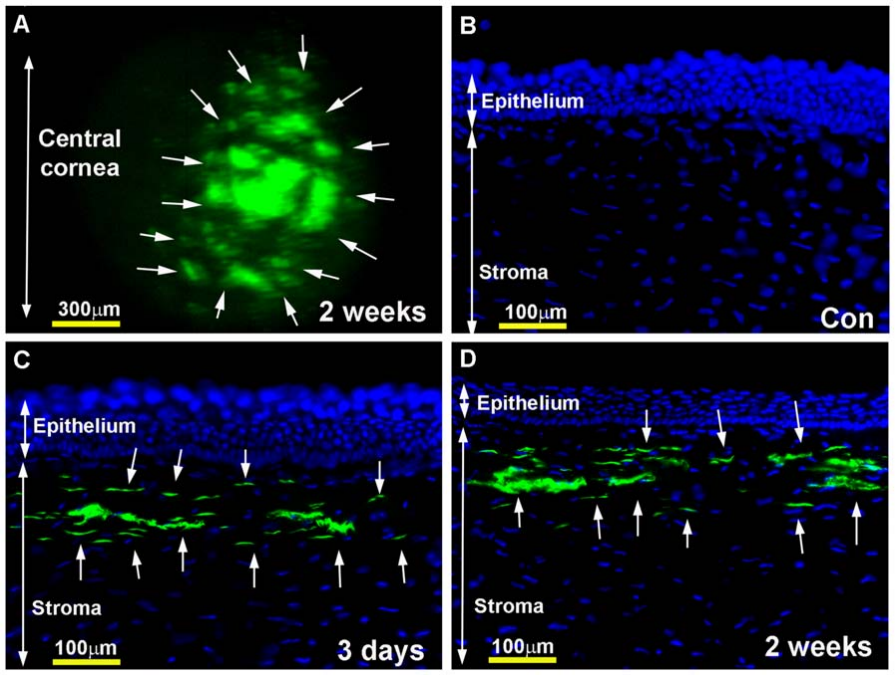
Representative *in vivo* fluorescence stereomicrograph (A) and tissue sections (B–D) of rabbit corneas showing AAV5-mediated GFP gene expression at 3-day and 2-week time points. Topical application of AAV5-GFP vector selectively transduced anterior keratocytes (arrows) located beneath the epithelium (C, D). No transgene expression was detected in control corneas (A). The rabbit corneas collected at 4-week and 16-week showed similar levels of GFP expression with immunostatining (data not shown). Nuclei are stained blue with DAPI. Scale bar denotes 100 µm.

Figure 1 shows AAV5-mediated delivery of GFP gene in the normal rabbit cornea detected using stereo- (Fig. 1A) and fluorescent- (Fig. 1B–D) microscopy. Rabbits were subjected to fluorescence imaging every 12 h for the first 3 days after AAV5 vector application, and thereafter once a week until euthanasia. All rabbit corneas showed initial appearance of GFP gene expression at day-3 (Fig. 1 C), which reached its maximum level at day-7. Fig. 1D shows a representative image of peak level of GFP expression in rabbit corneal tissue section detected at 2-week time point. No fluorescence was detected in corneas of early tested time points (12 h, 24 h, 48 h or 60 h). Rabbit corneas of later time points (2-week, 4-week and 16-week) showed fluorescence levels similar to the levels of 7-day time point. These observations suggest that AAV5 delivered transgene expression first appeared between 60 h to 72 h after vector application, continued to increase for the next 4 days, peaked at day-7, and maintained high transgene expression up to the longest tested time point of 16-week (4 months) in the rabbit corneas *in vivo*.

### Quantification of AAV5-mediated gene transfer

**Figure 2:**
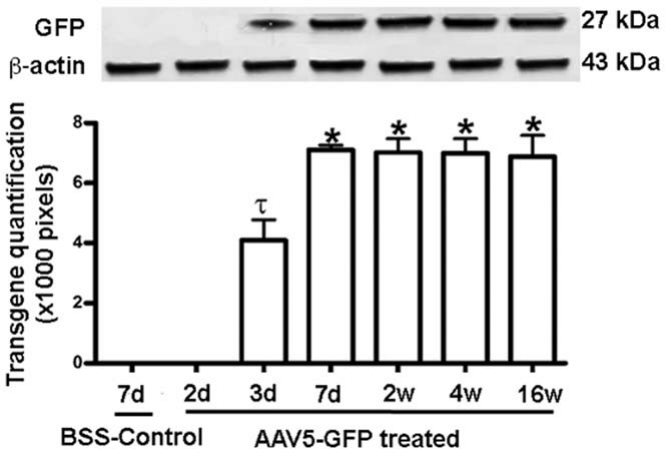
Representative western blot (upper panel) and digital quantification (lower panel) of delivered trangene in rabbit corneas at various time points. The delivered transgene expression first detected at 3-day, peaked at 7-day and maintained up to longest tested 16-week time point. β-actin was used to confirm equal loading of protein in each well and normalization of data. * p<0.05 compared to BSS-treated controls and 3-day time point, and τ p<0.01 compared to BSS-treated control.

The level of AAV5 delivered GFP gene expression was quantified using western blot. Figure 2 shows the levels of delivered GFP protein in rabbit corneas at various tested time points (2-day, 3-day, 7-day, 2-week, 4-week and 16-week) after single topical application of AAV5. The digital quantification of the western blot depicting the average pixels of three independent experiments is shown in Figure 2. The first detectable expression was noted at day-3 (4099 pixels±682). The maximum GFP expression was observed at day-7 (7100 pixels±154), which was significantly higher compared to day-3 (p<0.05) and balanced salt solution (BSS)-treated controls (p<0.01). Also, the GFP expression detected at other tested time points of 2-weeks (7021 pixels±462), 4-weeks (6998 pixels±473) and 16 weeks (6880 pixels±698) was significantly (p<0.05) higher than the GFP expression detected at day-3 as well as BSS-treated controls. No GFP expression was detected in BSS-treated control rabbit corneas. Equal loading of protein was confirmed by the detection of similar intensity β-actin bands.

### Spatial localization of AAV5-mediated GFP gene transfer

**Figure 3:**
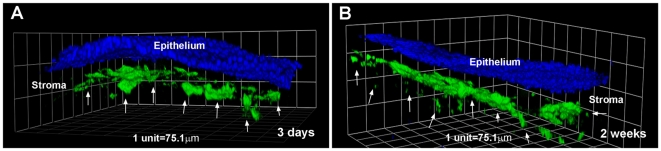
Representative three-dimensional confocal microscopy images showing spatial localization of GFP-expressing keratocytes (arrows) in whole-mount rabbit corneas exposed to AAV5. Corneas collected 3 days (A) and 2 weeks (B) after topical application of AAV5-GFP vector showed GFP-positive keratocytes beneath the epithelium in the anterior stroma. Nuclei are stained blue with DAPI. Scale bar denotes 75 µm.

To detect spatial localization of AAV5-mediated gene transfer in the rabbit cornea, we performed confocal microscopy in rabbit corneal tissues collected 3 days and 2 weeks after AAV5 application. The three-dimensional z-stack confocal images presented in Figure 3 reveal localization of GFP in rabbit corneas of 3-day (A) and 2-week (B) time points. As evident from this figure, the delivered-GFP gene expression was detected in the anterior stroma just below the corneal epithelium. No transgene expression was detected either in the corneal epithelium or posterior stroma or corneal endothelium. These observations suggest that AAV5 vector administered to the rabbit cornea with defined vector-delivery technique provided tissue-selective localized gene delivery in the anterior stroma.

### Determination of delivered-GFP gene copies with AAV5

**Figure 4:**
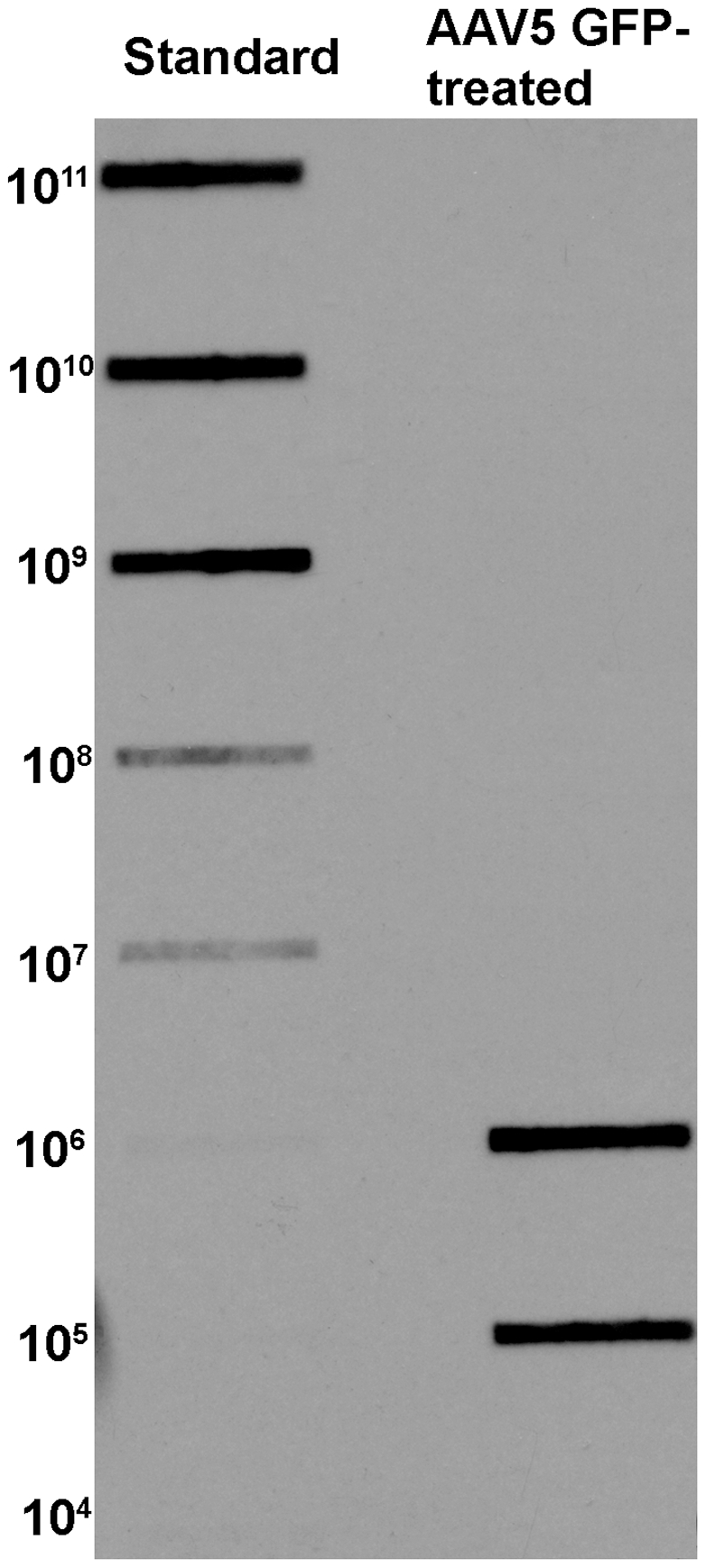
Slot blot showing GFP gene copy number in AAV5-GFP treated rabbit corneas. Densitometric comparison detected 10^8^–10^10^ copies of GFP gene in AAV5 GFP-treated rabbit corneas. Left lane shows standard plot of GFP plasmid DNA copies blotted at 10-fold dilution series. The right lane shows delivered GFP DNA copies detected in rabbit corneas collected 2-week after AAV5-GFP application.

To understand the correlation between delivered-GFP gene copy number and expression of delivered-GFP protein, we measured AAV5-delivered GFP gene copy number in rabbit corneas using slot blot. Figure 4 shows the gene copy number delivered in two separate rabbit corneas detected at 2-week time point using slot blot method. Densitometric analysis revealed that 10^8^–10^10^ genomic copies of transgene were detected in the rabbit corneas. This data complement the results of immunocytochemistry (Fig. 1) and western blotting (Fig. 2).

### AAV5-mediated gene delivery in diseased rabbit corneas

**Figure 5:**
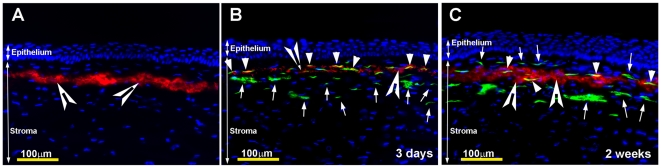
Representative images of corneal sections showing efficacy of AAV5-mediated transgene delivery in fibrotic rabbit corneas. Corneal fibrosis was produced by PRK laser surgery which induces transdifferentiation of keratocytes (myofibroblasts showing red staining for αSMA). Keratocytes expressing delivered GFP are shown in green (arrow), transdifferentiation keratocytes (myofibroblasts) expressing GFP and α-smooth muscle actin are shown in yellow (arrowheads), and transdifferentiation keratocytes (myofibroblasts) expressing α-smooth muscle actin are shown in red (Cut arrowheads). Nuclei are stained blue with DAPI. Scale bar denotes 100 µm.

Diseases affecting the corneas are associated with significant alterations in corneal homeostatic and/or cellular phenotype. Thus, we raised a question “do gene transfer parameters optimized using normal rabbit corneas are applicable for the diseased cornea?” To answer this question we used two most acceptable *in vivo* rabbit disease models; the PRK-based corneal scarring model and the VEGF-induced corneal neovascularization model to test the potential of optimized tissue-targeted gene transfer approaches using AAV5 for treating corneal diseases such as corneal fibrosis and corneal neovascularization. The gene transfer data observed in scarred rabbit cornea is shown in Figure 5. The detection of transdifferentiated keratocytes (myofibroblasts) with αSMA (a fibrosis biomarker) immunostaining (Fig. 5A) shown in red confirmed the scarring in rabbit corneas induced by PRK surgery. AAV5-delivered GFP gene expression (shown in green color) detected at 3-day (Fig. 5B) and 2-week (Fig. 5C) time points is shown in green. As evident from Figure 5B and 5C, AAV5 delivered significant levels of GFP in the anterior stroma of the scarred rabbit cornea. Furthermore, co-localization of GFP and αSMA (detected in yellow) suggests that transgene was also delivered into transdifferentiated keratocytes (myofibroblasts) in addition to keratocytes by this technique. These observations revealed that defined gene transfer parameters are efficient for corneal gene therapy.

**Figure 6:**
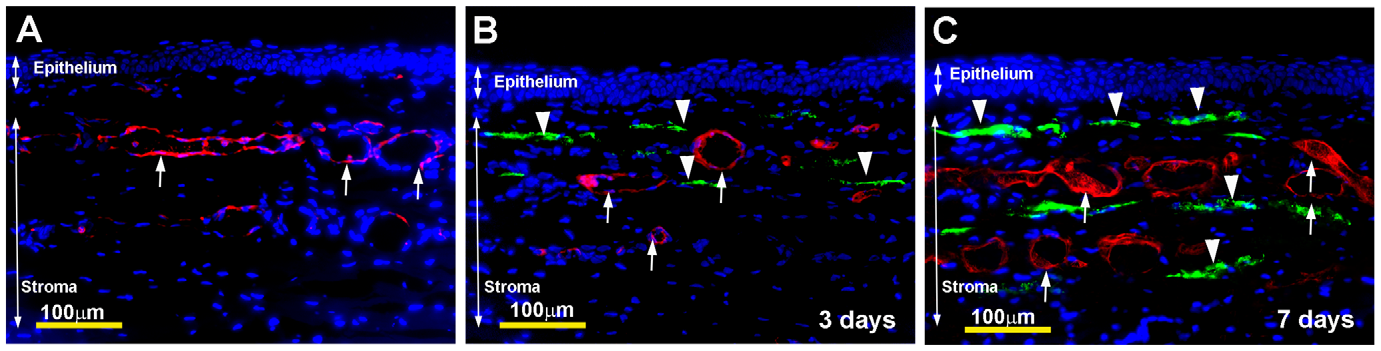
Representative *in vivo* images of corneal tissue sections showing efficacy of AAV5-mediated transgene delivery in neovascularized rabbit cornea. Keratocytes expressing delivered GFP are shown in green (arrowheads). Blood vessels are stained red with lectin (arrows). Nuclei are stained blue with DAPI. Scale bar denotes 100 µm.

Next, we evaluated the efficiency of defined gene transfer parameters using AAV5 for delivering genes into neovascularized rabbit corneas. Figure 6 shows that a single 2 minutes topical application of AAV5-GFP vector on the rabbit stroma delivered significant levels of transgene into neovascularized rabbit corneas further validated suitability of optimized parameters and tested AAV5 vector for delivering therapeutic genes in diseased corneas. The increased GFP expression detected at 7-day (Fig. 6C) compared to 3-day (Fig. 6B) time point suggests that neovascularization did not alter kinetics of AAV5-mediated gene transfer. GFP delivery and blood vessel formation in the rabbit corneal section in Figure 6 is shown with GFP immmunostaining with green and lectin staining in red, respectively.

### Safety determinations with slitlamp biomicroscopy and histology of AAV5-treated rabbit corneas

**Figure 7:**
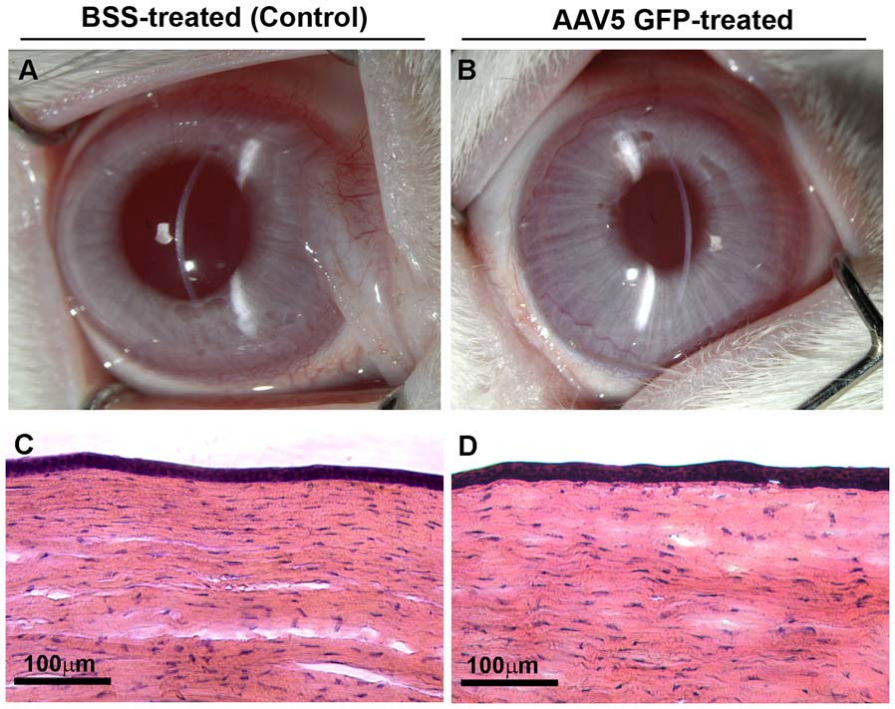
Representative slit lamp microscopy and images demonstrating safety of tested AAV5 to the cornea. No inflammation, redness, water discharge, swelling, etc was observed in BSS-treated control (A) or AAV5-treated corneas (B). Hematoxylin and eosin staining of corneal tissue sections (D) obtained from AAV5-treated rabbit eyes showed corneal morphology comparable to control corneas (C). Panels A–D shows data of 1-day time point. Similar observations were recorded for other tested time points (data not shown). Scale bar denotes 100 µm.

To analyze the effects of AAV5 on corneal health, visual and slit-lamp clinical examinations were performed in the eyes of live rabbits 1-day, 2-day, 3-day, 7-day and 4-week after BSS or AAV5 application. Neither BSS (Fig. 7A) nor AAV5-GFP (Fig. 7B) treated rabbit eyes showed inflammation, unusual discharge, swelling, redness or infection in the eye during clinical examination suggesting that AAV5 vector and used topical delivery technique are safe for the rabbit cornea. The hematoxylin and eosin-staining of BSS-treated control (Fig. 7C) and AAV5-treated rabbit corneas (Fig. 7D) collected at various time points did not exhibit any apparent structural abnormalities or abnormal infiltration of inflammatory cells in the rabbit cornea further confirming the safety of AAV5 vector for corneal gene delivery.

## Discussion

Gene therapy offers a novel opportunity to cure ocular surface disorders by targeting the underlying cause as opposed to simply treating the symptoms with conventional drug treatment. Safe and successful progression of gene therapy from bench-to-bedside application requires delivery of therapeutic genes into targeted tissue in a selective manner. The major reasons for the failure of gene therapy are the severe side effects because of the uncontrolled and untargeted delivery of therapeutic genes into tissues [5]. This study, for the first time, demonstrates site-selective targeted gene delivery into keratocytes of normal and damaged corneas *in vivo* using a rabbit model, and reports optimal conditions for achieving controlled and targeted expression of therapeutic genes in the cornea for treating blindness due to corneal disorders. Furthermore, our data demonstrate that AAV5 is an efficacious and safe vector for corneal gene therapy as a single two minute topical application of vector provided high levels of delivered-gene starting from day three and lasting over several months without causing significant side effects.

Corneal epithelium spans 5–7 cell layers and acts as a barrier to prevent entry of foreign particles and pathogens into the eye. We previously found that removal of corneal epithelium is a critical step for achieving high levels of transgene delivery in the stroma of mouse and rabbit corneas *in vivo* [21], [28], [29]. The removal of corneal epithelium via gentle scraping is a common clinical practice in the ophthalmology clinic to treat corneal epithelial defects, and its removal is a standard step in photorefractive keratectomy and laser epithelial keratomileusis surgeries. The healthy corneal epithelium regenerates within 24–72 hours after removal without causing any detrimental effects to the eye. Thus, to test our postulate that direct interaction of AAV to the bare stroma would provide enhanced gene delivery into keratocytes we took advantage of a common clinical practice of removing corneal epithelium, and topically applied AAV5 on de-epitheliazed corneas. Based on the lessons learned from our earlier studies that epithelial injury could lead keratocyte apoptosis in the anterior stroma and affect corneal healing, epithelial removal was carried-out by gentle scarping by advancing the blade from a 45° angle. This minor technique adjustment showed minimal apoptosis and depletion in keratocyte density in anterior stroma of the rabbit cornea. Topical application is the most acceptable method for delivering therapeutics to the eye and was therefore chosen for the study. However, topical dispensing not only renders contact of therapeutics to the cornea but also to other ocular tissues such as conjunctiva, sclera, iris, etc. To limit non-targeted ocular tissue transduction and maximize transgene delivery into keratocytes, a custom-cloning cylinder was used [29]. It has been our central hypothesis that localized and controlled administration of vector in the cornea via minimally invasive simple surgical techniques would allow targeted therapeutic gene delivery into desired cells of the cornea, *in vivo*. We used this approach for defining tissue-selective gene therapy approaches for the cornea because it does not require usage of a cornea-tissue specific promoter. At present, keratocan and the aldehyde dehydrogenase 3 cornea-specific promoter are generally used but both have their own limitations including leaky expression [30]–[32].

Corneal stroma is affected in many clinical disorders including corneal scarring, neovascularization, keratitis, graft rejection, ulcer and genetic dystrophies. Keratocytes residing in the stroma play an important role in maintaining corneal homeostasis, wound healing and clarity [33], [34]. Identification of efficacious and safe gene therapy approaches for the stroma has potential to lead to the development of novel therapeutic modalities for treating these corneal diseases and disorders [8], [12], [13]. It is well documented that during pathologic conditions keratocytes undergo phenotypic changes. This raises a question whether gene therapy methods optimized using normal corneas or healthy keratocytes could also be applicable to diseased conditions. In this study we addressed this issue by evaluating the efficiency of defined tissue targeted gene therapy approaches using two well-established animal disease models; rabbit corneal scarring and rabbit corneal neovascularization. In scarred rabbit corneas, AAV5 efficiently delivered GFP gene in the stroma and transduced a significant population of keratocytes as well as other cell types such as myofibroblasts whereas in neovascularized rabbit cornea transgene delivery was detected predominantly in keratocytes. On one hand, these findings confirmed that defined gene transfer approaches could efficiently deliver therapeutic genes to diseased corneas; on the other hand, the findings prompted us to raise another clinically relevant and important question “what would be a more effective therapeutic strategy: to deliver therapeutic gene into transdifferentiated keratocytes or normal keratocytes?” We postulate that it will largely depend on the selection of the therapeutic gene. For example, corneal scarring treatment with basic fibroblast growth factor (FGF2) gene would require targeted delivery of FGF2 gene into myofibroblasts (transdifferentiated keratocytes) as Maltseva et al. have shown transdifferentiation of myofibroblasts to keratocytes by FGF2 *in vitro* [35]. There is a possibility that FGF2 gene delivery into normal keratocytes could induce neovascularization in the cornea. This assumption is based on the fact that implantation of recombinant FGF2 pellet in the stroma is used to induce angiogenesis in the cornea [36], [37]. Our future studies will address such important questions.

Preclinical studies testing gene delivery in cornea have used a wide array of viral vectors. Multiple factors such as tissue tropism, duration of gene expression, vector gene carrying capacity, integrating or non-integrating nature of the vector, toxicity and safety of vector etc. dictate the choice of vector for clinical application of gene therapy. The safety and toxicity of vector to the patient and the environment remained a major determinant that enabled AAV to emerge as a vector of choice among viral vectors for ocular gene therapy. AAV is considered the safest viral vector because of its low immunogenic properties and non-pathogenicity to humans. The ocular gene therapy clinical trials carried-out with AAV serotype 2 (AAV2) for the retina have not reported any severe immune or inflammatory reactions among patients with used AAV vectors [38]–[40]. Like AAV2, AAV5 employed in this study did not show any significant side effects including the immune reaction or corneal damage suggesting that selected AAV5 is safe for corneal gene therapy. Scores of studies have demonstrated that AAV5 has superior transduction efficacy than AAV2 for ocular and non-ocular tissues [8], [38], [41]. Another factor that favors use of AAV5 for human gene therapy is the presence of AAV2 neutralizing antibodies in humans, which diminishes efficacy of AAV2 [42].

In summary, the site-selective controlled gene therapy approaches for the cornea defined using AAV5 vector and minimally invasive simple surgical technique may be effectively applied clinically to deliver genes in the eye to treat blindness from corneal stromal abnormalities. The AAV5 may potentially be a preferred vector for corneal gene therapy because of higher transduction efficiency and safety profile compared to AAV2.

## Materials and Methods

### Animals

The Institutional Animal Care and Use Committee of the University of Missouri-Columbia, Missouri USA USA (ID# 4279 and 6487) and Harry S. Truman Memorial Veterans' Hospital Columbia, Missouri USA (ID# 0041 and 0089) approved the study. Animals were treated in adherence to the principles of the ARVO Statement for the Use of Animals in Ophthalmic and Vision Research. New Zealand White rabbits (Myrtle laboratories Inc., Thompson's Station, TN) weighing 2.5–3.0 kg were used in this study. Rabbits were anesthetized by intramuscular injection of ketamine hydrochloride (50 mg/kg) and xylazine hydrochloride (10 mg/kg) for performing PRK, VEGF-implantation, stereo- and slit-lamp biomicroscopy.

### AAV5 vector generation

The AAV5 expressing green fluorescent protein gene (AAV5-GFP) titer produced at the Gene Therapy Vector Core Lab, University of Florida, Gainesville, Florida was procured from Prof. Gregory S. Schultz and Dr. Vince A. Chido. Following an earlier reported method the AAV2 plasmid pTRUF11 expressing fluorescent green protein gene under control of a hybrid promoter (cytomegalovirus enhancer and chicken β-actin) and simian virus 40 polyadenylation site was packaged into AAV5 [43]. In brief, AAV5 vector was produced by the 2-plasmid, co-transfection method. One Cell Stack (Corning Inc., Corning, NY, USA) with approximately 1×10^9^ HEK 293 cells was cultured in Dulbecco's Modified Eagle's Medium (Hyclone Laboratories, Inc. Logan UT, USA), supplemented with 5% fetal bovine serum and antibiotics. A CaPO_4_ transfection precipitation was set up by mixing a 1∶1 molar ratio of AAV2 plasmid DNA containing GFP and AAV5 rep–cap helper plasmid DNA. This precipitate was applied to the cell monolayer and the transfection was allowed to incubate at 37°C, 7% CO_2_ for 60 h. The cells were then harvested and lysed by freeze/thaw cycles and subjected to discontinuous iodixanol gradients centrifugation at 350,000 g for 1 h. This iodixanol fraction was further purified and concentrated by column chromatography on a 5-ml HiTrap Q Sepharose column using a Pharmacia AKTA FPLC system (Amersham Biosciences, Piscataway, NJ, USA). The vector was eluted from the column using 215 mM NaCl buffer, pH 8.0, and the rAAV peak collected. AAV5 GFP vector-containing fraction was then concentrated and buffer exchanged in Alcon BSS with 0.014% Tween 20, using a Biomax 100 K concentrator (Millipore, Billerica, MA, USA). Vector was titered for DNAse-resistant vector genomes by Real-Time PCR relative to a standard.

### AAV5 transduction to rabbit cornea

Twenty-eight rabbits ware used for the study. Only one eye of each rabbit selected randomly was used for the experiment. Sixteen rabbits were divided into two groups for the optimization of gene delivery parameters for the cornea. Rabbits of AAV5-treated group (n = 10) received 100 µl titer (6.5×10^12^ vg/ml) of AAV5 expressing green fluorescent protein gene under control of cytomegalovirus enhancer and chicken β-actin promoters topically for 2 minutes on de-epithelialized cornea via a custom hairdryer based vector delivery technique reported recently [29]. The control group (n = 6) received balance salt solution (BSS) topically using similar conditions. Twelve rabbits were used to evaluate the efficiency of optimized gene transfer parameters for delivering genes into diseased corneas namely rabbit corneal scarring model (n = 6) and rabbit neovascularization model (n = 6) were used. The AAV5 vector was topically applied to scarred rabbit cornea 4 weeks after PRK (n = 6) or neovascularized rabbit corneas 5-day after VEGF implantation (n = 6) using similar vector volume, titer, delivery technique, and experimental conditions. The contralateral eyes served as a naive control.

### Corneal neovascularization and haze generation

Neovascularization in rabbit cornea was induced by corneal micro-pocket assay [44]. Rabbits were anesthetized with ketamine and xylazine, and 3–4 drops of 0.5% topical proparacaine hydrochloride solution (Alcon, Ft. Worth, TX, USA) was applied to the eye prior to cornea micropocket surgery. Only one eye of each animal was used for surgical procedure. The contralateral eye served as naive control. A wire speculum was positioned in the eye and a sucralfate-hydron pellet containing 650 ng of VEGF (PeproTech, Rocky Hill, NJ) was implanted into the cornea after making a micropocket in the cornea using standard surgical tools. Triple antibiotic ointment (Alcon) was applied to the surface of the cornea after pellet implantation to prevent infection. The ingrowth of blood vessels in the cornea towards the VEGF implant started from day 2, peaked around day 10 and continued to grow progressively up to 15 days before regressing.

Haze in rabbit cornea was produced by performing photorefractive keratectomy (PRK) surgery in an anaesthetized rabbit [45]. Topical proparacaine hydrochloride 0.5% (Alcon, Ft. Worth, TX, USA) was applied to each eye just before PRK. A wire lid speculum was positioned and a 7 mm-diameter area of epithelium overlying the pupil was removed by scraping with a #64 blade (Beaver; Becton-Dickinson, Franklin Lake, NJ, USA). The −9.0 diopter PRK surgery with a 6 mm ablation zone on the central stroma was performed using the Summit Apex excimer laser (Alcon, Ft. Worth, TX). Only one eye from each animal was used for PRK and the contralateral eye served as naive control. The corneal haze in animals peaked 4 weeks after PRK.

### Clinical and slit-lamp biomicroscopy

The health of the cornea in eyes of live rabbits was examined by visual clinical and slit-lamp microscopic (BX 900 Slit Lamp, Haag-Streit-USA, Mason OH) examinations before and after AAV5 application in normal and diseased (hazy or neovascularized) rabbit corneas by two ophthalmologists and a researcher, independently and in a blinded manner while animals were under general anesthesia. Thereafter, corneal health was monitored every third day with a hand-held slit-lamp microscope (SL-15, Kowa Optimed Inc., Torrance, CA). Photographs of the cornea were taken with a digital camera attached to the BX 900 slit-lamp microscope.

### Tissue collection

Rabbits were humanely euthanized with pentobarbitone (150 mg/kg) overdose under general anesthesia at selected time points. Rabbit corneas were removed with forceps and sharp Westcott scissors and cut into 2 equal halves. One half was embedded in liquid optimal cutting temperature (OCT) compound (Sakura FineTek, Torrance, CA) within a 24 mm×24 mm×5 mm mold (Fisher, Pittsburgh, PA) and snap frozen. Frozen tissue blocks were maintained at −80°C. Tissue sections were cut 7 µm thick with a cryostat (HM 525 M, Microm GmbH, Walldorf, Germany). Sections were placed on 25 mm×75 mm×1 mm microscope Superfrost Plus slides (Fisher), and maintained frozen at −80°C until staining. The other half of rabbit corneal tissues was snap frozen directly in liquid nitrogen for isolating RNA, DNA or protein.

### Immunohistochemistry and hematoxylin and eosin staining

Corneal tissues were stained with hematoxylin and eosin (H & E). Immunofluorescence staining for alpha smooth muscle actin (αSMA), a marker for myofibroblasts, was performed using mouse monoclonal primary αSMA antibody (1∶200 dilution, catalog no. M0851, Dako, Carpinteria, CA). Tissue sections were incubated with 2% bovine serum albumin for 30 minutes at room temperature and then with αSMA monoclonal antibody for 90 minutes. For the detection of the primary antibody, Alexa 488 goat anti-mouse IgG secondary antibody (1∶1000 dilution; catalog no. A11001, Invitrogen Inc., Carlsbad, CA) for 1 hour was used.

Blood vessel formation was confirmed with tomato lectin staining which entailed the incubation of corneal sections with 20 µg/ml Texas red-conjugated tomato lectin (cat # TL-1176; Vector laboratories, Burlingame, CA) for 90 min. Tissue sections were washed in HEPES buffer and mounted using Vectashield medium containing 4′-6-diamidino-2-phenylindole (DAPI; Vector laboratories). The stained sections were viewed and photographed with a Leica fluorescent microscope (Leica DM 4000B; Leica) equipped with a digital camera (SpotCam RT KE).

### Immunoblotting

Protein lysates were prepared by homogenizing corneas in protein lysis buffer containing protease inhibitor cocktail (Roche Applied Sciences, Indianapolis, IN). Total protein was determined with Bradford assay. The same amount of protein of each sample was suspended in Laemmli denaturing sample buffer, vortexed and heated for 10 min at 70°C. The proteins were resolved on 4–20% SDS-PAGE gel and transferred onto 0.45 µm pore size PVDF membrane (Invitrogen, San Diego, CA). The membrane was incubated with GFP (cat # sc-33856; Santa Cruz) or β-actin (cat # sc-69879; Santa Cruz) primary antibody followed by alkaline phosphatase-conjugated anti-goat or anti-mouse secondary antibody (Santa Cruz). The bands were visualized by NBT/BCIP.

### Stereo-biomicroscopy and confocal microscopy

Fluorescent stereomicroscope (model MZ16F, Leica) was used to track GFP expression in the eye of live rabbits under general anesthesia. The spatial localization of delivered-GFP gene in whole-mounts of normal cornea and thick tissue sections of damaged corneas was determined with confocal microscope (TCS-SP; Leica or Radiance 2000; Bio-Rad) using corresponding lasers for DAPI and GFP. The paraformaldehyde (4%) fixed corneal whole-mount tissues were stained with DAPI for 3 days to stain nuclei. The 20 µm thick corneal sections of the damaged rabbit corneas were subjected to triple staining (nuclei with DAPI in blue, cells expressing-GFP in green, and cells expressing SMA or lectin with red). The Z-stacks were generated in 0.45 µm increments and 3-D reconstructions were created by computer using Velocity software (Impro Vision Inc., Lexington, MA). The 3-D images were rotated 360° for spatial and perceptual visualization of the corneal regions. The exact location and quantity of the EGFP-positive cells in the cornea were measured with Velocity software (Impro Vision) and NIH Image J software.

### Slot blot analysis

The copies of delivered plasmid were determined with slot blot analysis. Frozen corneal tissues were ground in liquid nitrogen and DNA was isolated using the DNA easy kit (Qiagen, cat # 69504). The standards were prepared using 10^4^–10^11^ copies of decorin gene cloned into pTRUF11 vector. The DNA probe was prepared by digesting 5 µg of decorin plasmid with Not1 restriction enzyme and labeling 1 µg of isolated decorin fragment with digoxigenin (DIG)-labeled UTP, using DIG starter Kit II (catalog no. 11585614910 Roche Applied Science, Indianapolis, IN). Two microliters of the standard as well as the DNA isolated from corneal tissues was denatured by alkali and heat treatment. Denatured DNA samples were blotted onto nylon membrane using slot blot apparatus (BioRad lab) and were UV-cross linked. The membrane was hybridized with 300 ng of digoxigenin (DIG)-labeled probe overnight at 30°C, followed by incubation in 1∶5000 anti-digoxigenin-AP antibody. Chemiluminiscent detection was used following vendor's instructions (catalog no. 11585614910 Roche Applied Science, Indianapolis, IN) and membrane was exposed to X-ray film. Image J 1.38× image analysis software was used to determine delivered gene copies in samples by measuring dot intensities of samples and comparing the data with standards.
